# Biopsychosocial factors associated with disordered eating behaviors in schizophrenia

**DOI:** 10.1186/s12991-020-00314-2

**Published:** 2020-11-27

**Authors:** Mohsen Khosravi

**Affiliations:** Department of Psychiatry and Clinical Psychology, Baharan Psychiatric Hospital, Zahedan University of Medical Sciences, 9813913777 Zahedan, Iran

**Keywords:** Behavior, Eating, Schizophrenia

## Abstract

**Background:**

Recent hypotheses have suggested that schizophrenic patients are more likely to consume unhealthy foods, causing increased rates of mortality and morbidity associated with metabolic syndrome. This raises the need for more in-depth research on disordered eating behaviors (DEBs) in schizophrenic patients. This study, therefore, aimed to investigate biopsychosocial factors associated with DEBs in schizophrenia.

**Methods:**

In this cross-sectional study, a total of 308 participants (including 83 subjects in the active phase of schizophrenia, 71 subjects in the remission phase of schizophrenia, and 154 control subjects) were recruited through convenience sampling among patients who referred to the Baharan Psychiatric hospital in Zahedan, Iran. Patients were assessed through Eating Attitudes Test (EAT-26), Beck Anxiety Inventory (BAI), Beck Depression Inventory (BDI-II), and Positive and Negative Syndrome Scale (PANSS). Data were analyzed using SPSS v25 software. Further, the statistical significance level was set at *p* < 0.05.

**Results:**

The prevalence of DEBs was 41.5% in schizophrenic patients (vs. 10.3% in the control group, *p* = 0.012). No significant difference was observed in the EAT-26 scores based on gender and phases of schizophrenia. According to multiple linear regression analysis, lack of psychosocial rehabilitation, use of atypical antipsychotics, early stages of psychosis, high level of anxiety and depression, expression of more active psychotic symptoms, tobacco smoking, and suffering from type 2 diabetes were all associated with increased development of DEBs among schizophrenic patients.

**Conclusions:**

Since the occurrence of DEBs is independent of different phases of schizophrenia, the risk of DEBs is required to be evaluated during the entire course of schizophrenia especially at earlier stages of schizophrenia. Moreover, the use of psychosocial interventions, treatment of affective disorders (i.e., anxiety and depression), antipsychotic medication switching, treatment of tobacco smoking and type 2 diabetes may reduce the risk of DEBs among schizophrenic patients. However, further investigations are required to prove the actual roles of the above factors in developing DEBs among schizophrenic patients.

## Background

As a convoluted topic, disordered eating behaviors (DEBs) need to be recognized and defined [1]. The manifestations of DEBs may include behaviors such as binge eating, food restriction, and purging that are commonly related to feeding and eating disorders (FEDs) [2]. Nevertheless, these symptoms are less frequent or at a lower level of severity and might not be as extreme as symptoms of diagnosable FEDs [1, 2]. Furthermore, subjects who exhibit these behaviors may be at risk, both physically and emotionally [2].

Recent hypotheses have implied that schizophrenic patients more probably consume unhealthy foods, provoking escalated rates of mortality and morbidity attributed to metabolic syndrome [3]. This emphasizes the need for further in-depth research into DEBs in schizophrenic patients. Patients with DEBs are often characterized by a lack of control over their eating behaviors, known as “voracious gorging”, or as a form of environmental automatism [2, 3]. Despite the introduction of disorganized and uncontrolled food intake by Kraepelin [4] and Bleuler [5] as one of the characteristics of schizophrenia in the nineteenth century, the literature on this problem is still scarce because no sole criterion has been presented to provide a definite diagnosis of DEBs [6–8]. This has caused DEBs to be a secondary concern for clinicians [9]. In this respect, a recent hypothesis has suggested that DEBs may be a physiological compensatory mechanism in reducing psychotic symptoms, as starvation induces psychosis in individuals [3]. The most famous illustration of starvation-induced psychosis is related to the Minnesota study, wherein two healthy male volunteers showed similar behaviors following 24 weeks of starvation to patients with schizophrenia and anorexia nervosa [3].

Although various studies have focused on schizophrenia with comorbidity of FEDs such as anorexia nervosa, bulimia nervosa, binge eating, and other specified feeding and eating disorders (especially night eating syndrome), the pathophysiology of DEBs in schizophrenia has remained unknown [6, 10]. Depression and/or anxiety, sleep disturbances, socioeconomic adversity, side effects of psychiatric medications, tobacco smoking, and type 2 diabetes have been believed to be the risk factors for DEBs in schizophrenic patients [11]. Consistent with these findings, recent studies have revealed that over 70% of individuals with DEBs have a history of depression and/or anxiety before or after the onset of problematic eating behaviors [2, 12]. Bruch [13], for instance, considered overeating as an adaptive defense against stress among schizophrenic patients, which is utilized for the maintenance of self-control. She claimed that heavier people would not develop psychosis and used Kallman’s [14] monozygotic twin study argument to support this hypothesis. However, these early descriptions fail to explain cognitive awareness or deliberate behavior.

Additionally, recent animal and clinical researches have suggested that antipsychotics can lead to changes in dietary habits and hyperphagic effects, thereby causing a lack of satiation and increased appetite [15–17]. This evidence is consistent with previous research on schizophrenia patients, indicating that the treatment with atypical antipsychotics is associated with more frequent DEBs compared to typical antipsychotics [18–21]. In this regard, Sallemi et al. [16] used the SCOFF questionnaire to examine 53 Tunisian patients and reported DEBs among 35.8% of participants (mostly women and those with the use of atypical neuroleptics). Besides, Sentissi et al. [21] evaluated eating behaviors of 153 schizophrenic patients using the Three-Factor Eating Questionnaire (TFEQ) and reported that DEBs in patients are influenced by gender and treatment with atypical antipsychotics. On the other hand, no correlation was observed between medication use, duration of psychosis, and TFEQ scores in the study conducted by Kouidrat et al. [22] on 66 French schizophrenic patients. Also, their findings revealed no significant difference in TFEQ mean scores between male and female adults.

Moreover, comorbid medical and tobacco smoking are prevalent in schizophrenic patients [23]. For example, the risks of tobacco smoking and type 2 diabetes development in these patients were reported 58–90% [23] and 23.9% [24], respectively. In this point, recent evidence has suggested that tobacco smoking and type 2 diabetes may be associated with DEBs in schizophrenic patients [25, 26], e.g., DEBs in approximately 40% of schizophrenic patients with type 2 diabetes [26].

Despite these few and scattered findings, no study has yet comprehensively and specifically examined the biopsychosocial factors in DEBs among schizophrenic patients. Since DEBs occurrence in schizophrenic patients is an acute problem that accounts for the development of FEDs, complicate weight loss efforts, obesity, cardiometabolic disorders and their risk factors need to be identified for developing effective prevention and treatment strategies [1, 2, 6, 22]. Thus, the present study aims to investigate the biopsychosocial factors in DEBs among schizophrenic patients.

## Methods

### Participants

This cross-sectional study was conducted from May 2018 to November 2019. Based on the Green’s method [27], a total of 154 patients with schizophrenia (83 subjects in the active phase and 71 subjects in the remission phase) were selected by convenience sampling method among the people who referred to Baharan psychiatric hospital in Zahedan, Iran. Also, the control group was recruited from residents of the same geographical area through one-to-one matching (case:control ratio of 1:1; *n* = 154). The inclusion criteria were as follows: (1) a diagnosis of schizophrenia based on Structured Clinical Interviews for DSM-5 (Diagnostic and Statistical Manual of Mental Disorders, 5th Edition): Research Version (SCID-5-RV) by a psychiatrist; (2) aged between 18 and 70; (3) for the control group, getting a score of   < 21 in the 28-item General Health Questionnaire (GHQ-28) and approved mental health based on SCID-5-RV by the psychiatrist, except evidences for DEBs and FEDs which were not exclusion criterion. Exclusion criteria were specified as follows: (1) intellectual disability; (2) a history of neurological disorder; (3) the use of antidepressants or mood stabilizers in the previous 3 months; (4) hearing loss; (5) failing to fill the questionnaires properly. The socio-demographic information of the participants is presented in Table 1 (*N* = 308).

**Table 1:** Compression of three study groups based on socio-demographic variables (*N* = 308)

Variables	Categories	Active phase group (*n* = 83)	Remission phase group (*n* = 71)	Control group (*n* = 154)	Test^a^
		*n* (%)	*n* (%)	*n* (%)	
Age	20–29	24 (28.9)	24 (33.8)	43 (27.9)	*χ*^2^ = 2.63
	30–39	18 (21.7)	19 (26.8)	31 (20.1)	
	40–49	26 (31.3)	16 (22.5)	42 (27.3)	
	50–60	15 (18.1)	12 (16.9)	38 (24.7)	
Gender	Male	36 (43.4)	28 (39.4)	84 (54.5)	*χ*^2^ = 5.44
	Female	47 (56.6)	43 (60.6)	70 (45.5)	
Marital status	Married	42 (50.6)	35 (49.3)	88 (57.1)	*χ*^2^ = 1.60
	Single	41 (49.4)	36 (50.7)	66 (42.9)	
Residence	Private home	80 (96.4)	66 (93.0)	154 (100)	*χ*^2^ = 9.99**
	Homeless	3 (3.6)	5 (7)	0 (0.0)	
Educational level	Illiterate	17 (20.5)	20 (28.2)	40 (26.0)	*χ*^2^ = 1.23
	Elementary grade	16 (19.3)	11 (15.5)	29 (18.8)	
	Middle grade	13 (15.7)	14 (19.7)	23 (14.9)	
	High school	22 (26.5)	17 (23.9)	34 (22.1)	
	College	15 (18.1)	9 (12.7)	28 (18.2)	

^a^Statistical analyses applied Chi-square test and Kruskal–Wallis test

* *p* < 0.05; ** *p* < 0.01; *** *p* < 0.001

### Procedures

After obtaining the ethical approval from the Research Center of the Medicine Faculty and prior permission from the relevant Ethics Committee with IR.ZAUMS.REC.1398.210 code of ethics, the subjects were given the consent form to sign. The study was performed in compliance with the declaration of Helsinki, i.e., subjects were told that their participation would be optional and they could leave the study for any reason. After obtaining informed consent from the participants, the psychiatrist evaluated all of the participants using GHQ-28 and SCID-5-RV to identify the three study groups (including active phase group, remission phase group, and control group). Next, the 26-item Eating Attitudes Test (EAT-26), Beck Anxiety Inventory (BAI), Beck Depression Inventory (BDI-II), and Positive and Negative Syndrome Scale (PANSS) questionnaires were given to them. Then, schizophrenic patients with DEBs (i.e., earning an EAT-26 score of ≥ 20) were evaluated in terms of levels of anxiety and depression, type 2 diabetes (according to fasting blood sugar (FBS) ≥ 136 mg/dL on two separate tests), tobacco smoking (yes/no), duration of psychosis (i.e., from 6 months to 2 years, 2 to 5 years, 5 to 10 years, and greater than or equal to 10 years), phases of schizophrenia (active or remission phases), severity of psychosis, and category of antipsychotic medications (typical or atypical). The questionnaires were anonymous to preserve the participants’ information confidential.

## Measures

### EAT-26

A Persian version of the EAT-26 was used to measure DEBs among participants. The EAT-26 is a 26-item questionnaire that comprises questions on dieting, bulimia and food preoccupation, and oral control. The questions No. 1 to 25 were scored on a 6-point Likert scale, as 0 = never, 0 = rarely, 0 = sometimes, 1 = often, 2 = usually, and 3 = always. The only reverse-scoring item was question 26. The questionnaire scores may vary between 0 and 78, where a total score of ≥ 20 in the survey represents DEBs. A Cronbach’s alpha of 0.75 was obtained for this questionnaire, implying that this measure had acceptable validity and reliability [28]. In this study, the Cronbach’s alpha coefficient for the EAT-26 was 0.80.

### BAI

Anxiety symptoms were assessed with the Persian version of the BAI, which is a self-report 21-item questionnaire scored based on a four-point (0 to 3) Likert scale. The minimum and maximum scores are 0 and 63, respectively. Kaviani et al. [29] reported acceptable reliability and validity for the Persian version of the questionnaire (Cronbach’s alpha = 0.92). In this study, the Cronbach’s alpha coefficient for the BAI was 0.90.

### BDI-II

Depressive symptoms were assessed with the Persian version of the BDI-II, which is a self-report 21-item questionnaire scored based on a four-point (0 to 3) Likert scale. The minimum and maximum scores are 0 and 63, respectively. Ghassemzadeh et al. [30] showed acceptable reliability and validity for the Persian version of the questionnaire (Cronbach’s alpha = 0.87). In this study, the Cronbach’s alpha coefficient was 0.88 for the BDI-II.

### PANSS

Symptoms severity of schizophrenic patients was assessed with the Persian version of the PANSS, which is a 30-item questionnaire scored based on a 5-point Likert scale (1 = absent, 2 = minimal, 3 = moderate, 4 = severe, and 5 = extreme). The scores of the items were summed up, resulting in the minimum and maximum scores of 30 and 150, respectively. Cronbach’s alpha of this scale was estimated at 77%, and its validity was approved based on the factor analysis results [31]. In this study, the Cronbach’s alpha coefficient was 0.75 for the PANSS.

### SCID-5-RV

SCID-5-RV is a semi-structured interview for major DSM-5 diagnoses, which is performed by a trained clinician or health expert familiar with the diagnostic criteria and classification of disorders in DSM-5. Several studies have reported acceptable reliability and validity of SCID-5-RV [32].

### GHQ-28

GHQ-28 is a 28-item questionnaire wherein items are scored on a 0 to 3 scale. The overall scores range between 0 and 84. A score of ˂ 21 indicates a person’s mental health [33]. In this study, the Cronbach’s alpha coefficients for the GHQ-28 subscales of somatic symptoms, anxiety and insomnia, social dysfunction, and severe depression were 0.75, 0.75, 0.70, and 0.85, respectively, while it was 0.88 for the total scale.

### Statistical analysis

Descriptive statistics were obtained for data analysis. The Chi-square test and Kruskal–Wallis test were performed for a socio-demographic comparison among the study groups. Moreover, independent *t*-test and analysis of variance (ANOVA) were carried out to compare the EAT-26 scores among schizophrenic patients with DEBs based on biopsychosocial factors. Also, ANOVA was used to compare outcomes’ mean scores of EAT-26, BAI, and BDI-II between three study groups. In ANOVA, the Scheffé test was applied to post hoc analysis. The point-biserial correlation coefficient, Pearson correlation coefficient, and Spearman’s rank correlation coefficient were used to assess the correlations between DEBs and biopsychosocial factors among schizophrenic patients with DEBs (i.e., getting an EAT-26 score of ≥ 20). The multiple linear regression analysis was performed to examine the linear relationship between the response (i.e., DEBs) and explanatory variables (i.e., biopsychosocial factors). Data were analyzed using SPSS v25 software, and the statistical significance level was set at *p* < 0.05.

## Results

### Preliminary analysis

**Fig. 1:**
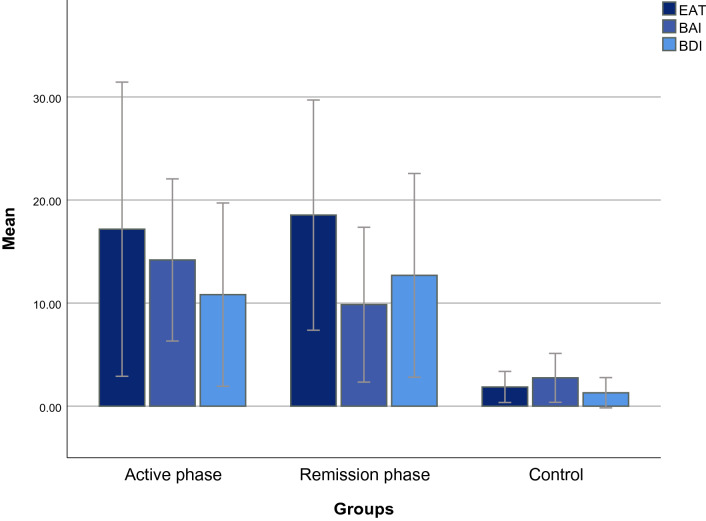
Clustered bar mean of Eating Attitudes Test (EAT-26), Beck Anxiety Inventory (BAI), and Beck Depression Inventory (BDI-II) by groups (error bars: 95% CI, ± 1 SD; *N* = 308). Analysis of variance (ANOVA). EAT-26: *F*(2, 305) = 116.14, *p* < 0.001; post hoc test: active phase, remission phase > control. BAI: *F*(2, 305) = 116.84, *p* < 0.001; post hoc test: active phase > remission phase > control. BDI-II: *F*(2, 305) = 94.26, *p* ˂ 0.001; post hoc test: active phase, remission phase > control

The DEBs were observed in 41.5% (*n* = 64) of schizophrenic patients (vs. 10.3% in the control group, *p* = 0.012), using the EAT-26. There was no significant difference in the EAT-26 scores regarding gender (*t*(62) = −  1.20, *p* = 0.231) and the phases of schizophrenia (*t*(62) = 1.16, *p* = 0.250). However, there were large differences in the EAT-26 scores concerning psychosocial rehabilitation (t(62) = −  3.43, *p* = 0.001), duration of psychosis (*F*(3, 60) = 11.34, *p* < 0.001), type of antipsychotic medications (*t*(42.20) = −  7.42, *p* < 0.001), tobacco smoking (*t*(16.90) = −  3.25, *p* = 0.005), and type 2 diabetes (*t*(14.16) = −  4.26, *p* = 0.001), as shown in Table 2. Comparisons of mean EAT-26, BAI, and BDI-II scores revealed significant differences among the three study groups including active phase group, remission phase group, and control group (*F*(2, 305) = 116.14, *p* < 0.001; *F*(2, 305) = 116.84, *p* < 0.001; *F*(2, 305) = 94.26, *p* < 0.001, respectively), such that the active phase group accounted for the highest scores of BAI (Fig. 1).

**Table 2:** Compression of the 26-item Eating Attitude Test (EAT-26) scores among schizophrenic patients with DEBs (i.e., earning an EAT-26 score of ≥ 20, *n* = 64) based on biopsychosocial factors

Variables	Categories	EAT-26 (total score)	Test^a^
		M ± SD (range)	
Age	20–29	29.89 ± 6.91 (22–49)	*F* = 2.58
	30–39	28.62 ± 7.16 (22–49)	
	40–49	35.66 ± 8.08 (29–49)	
	50–60	34.14 ± 10.77 (22–49)	
Gender	Male	29.35 ± 8.05 (22–49)	*t* = − 1.20
	Female	31.93 ± 7.86 (22–49)	
Marital status	Married	28.26 ± 7.17 (22–49)	*t* = − 1.91
	Single	32.33 ± 8.02 (22–49)	
Residence	Private home	31.35 ± 8.08 (22–49)	*t* = 0.87
	Homeless	27.75 ± 5.05 (22–32)	
Educational level	Illiterate	33.90 ± 9.17 (22–49)	*F* = 1.12
	Elementary grade	30.83 ± 9.80 (22–49)	
	Middle grade	28.18 ± 7.61 (22–49)	
	High school	30.33 ± 4.69 (22–38)	
	College	30.07 ± 7.40 (22–49)	
Psychosocial rehabilitation	With	28.43 ± 6.58 (22–49)	*t* = − 3.43**
	Without	34.81 ± 8.28 (22–49)	
Duration of psychosis	6 months to 2 years	36.56 ± 9.96 (22–49)	*F* = 11.34*** Post hoc test: 3, 4 < 1 4 < 2
	2 to 5 years	32.15 ± 1.90 (31–38)	
	5 to 10 years	28.33 ± 5.19 (22–38)	
	Higher than 10 years	25.15 ± 2.31 (22–29)	
Category of antipsychotic medications	Typical	25.61 ± 3.01 (22–31)	*t* = − 7.42***
	Atypical	36.30 ± 7.66 (22–49)	
The phases of schizophrenia	Active phase	32.17 ± 8.45 (22–49)	*t* = 1.16
	Remission phase	29.86 ± 7.22 (22–49)	
Tobacco smoking	With	38.12 ± 11.11 (22–49)	*t* = − 3.25**
	Without	28.79 ± 4.79 (22–38)	
Type 2 diabetes	With	40.38 ± 9.41 (25–49)	*t* = − 4.26**
	Without	28.76 ± 5.51 (22–49)	

* *p* < 0.05; ** *p* < 0.01; *** p < 0.001

^1^6 months to 2 years; ^2^2 to 5 years; ^3^5 to 10 years; ^4^Higher than 10 years

^a^Statistical analyses applied independent t-test, analysis of variance (ANOVA), and Scheffé post hoc test

### The correlations between DEBs and biopsychosocial factors

Significant correlations were found between DEBs (EAT-26 score of ≥ 20) and psychosocial rehabilitation (*r* = 0.40, *p* = 0.001), duration of psychosis (*r* = − 0.59, *p* < 0.001), category of antipsychotic medications (*r* = 0.67, *p* < 0.001), anxiety (*r* = 0.54, *p* < 0.001), depression (*r* = 0.48, *p* < 0.001), severity of psychosis (*r* = 0.53, *p* < 0.001), tobacco smoking (*r* = 0.51, *p* < 0.001), and type 2 diabetes (*r* = 0.59, *p* < 0.001), as can be seen in Table 3.

**Table 3:** Correlations between the disordered eating behaviors (DEBs) and biopsychosocial factors among schizophrenic patients with DEBs (i.e., earning a 26-item Eating Attitude Test score of ≥ 20; *n* = 64)

Explanatory variables	1	2	3
Age	–	–	0.20
Gender	0.15	–	–
Marital status	0.23	–	–
Residence	− 0.11	–	–
Educational level	–	–	− 0.17
Psychosocial rehabilitation	0.40**	–	–
Duration of psychosis	–	–	− 0.59***
The phases of schizophrenia	− 0.14	–	–
Category of antipsychotic medications	0.67***	–	–
Anxiety	–	0.54***	–
Depression	–	0.48***	–
Severity of psychosis	–	0.53***	–
Tobacco smoking	0.51***	–	–
Type 2 diabetes	0.59***	–	–

^1^Point-biserial correlation coefficient

^2^Pearson correlation coefficient

^3^Spearman’s rank correlation coefficient

* *p* < 0.05; ** *p* < 0.01; *** p < 0.001

### Biopsychosocial factors associated with DEBs among schizophrenic patients

According to multiple linear regression analysis, the biopsychosocial factors associated with DEBs among schizophrenic patients included psychosocial rehabilitation (*β* = 0.15, *p* = 0.042), duration of psychosis (*β* = − 0.18, *p* = 0.022), category of antipsychotic medications (*β* = 0.17, *p* = 0.045), anxiety (*β* = 0.16, *p* = 0.043), depression (*β* = 0.23, *p* = 0.003), severity of psychosis (*β* = 0.14, *p* = 0.047), tobacco smoking (*β* = 0.24, *p* = 0.007), and type 2 diabetes (*β* = 0.18, *p* = 0.035; *F*(8, 55) = 28.19, *R*^2^ = 0.80, *p* < 0.001), as presented in Table 4.

**Table 4:** Summary of regression analysis to determine the biopsychosocial risk factors for disordered eating behaviors (DEBs) among schizophrenic patients with DEBs (i.e., a 26-item Eating Attitude Test score of ≥ 20, *n* = 64)

Explanatory variables	*B* (*β*)	SE	*t*	95% CI	
				Lower bound	Upper bound
Psychosocial rehabilitation (yes)	2.44 (0.15)*	1.17	2.08	0.09	4.80
Duration of psychosis	− 1.17 (− 0.18)*	0.50	− 2.35	− 2.17	− 0.17
Category of antipsychotic medications (atypical antipsychotic)	2.78 (0.17)*	1.35	2.05	0.06	5.49
Anxiety	0.12 (0.16)*	0.06	2.07	0.00	0.24
Depression	0.17 (0.23)**	0.05	3.15	0.06	0.27
Severity of psychosis	0.13 (0.14)*	0.06	2.03	0.00	0.27
Tobacco smoking (yes)	4.40 (0.24)**	1.56	2.80	1.25	7.54
Type 2 diabetes (yes)	3.69 (0.18)*	1.70	2.16	0.27	7.12
*R*	0.89				
*R*^2^	0.80				
Adj. *R*^2^	0.77				
*F* (d*f*1, d*f*2)	28.19 (8, 55)***				

* p < 0.05; ** p < 0.01; *** p < 0.001

## Discussion

The findings of the present study can be divided into five major parts. As the first part, the DEBs prevalence was 41.5% among schizophrenic patients (vs. 10.3% in the control group), using the EAT-26. However, the prevalence of DEBs among schizophrenic patients was 35.8% in the study by Sallemi et al. [16] through the SCOFF questionnaire, and 30% in the study conducted by Fawzi et al. [34] using the 40-item Eating Attitudes Test (EAT-40). The disparity in the frequency of DEBs between the present study and the studies by Sallemi et al. [16] and Fawzi et al. [34] may be due to the type of questionnaires, the number of participants, socio-demographic characteristics, and disregarding schizophrenic patients with type 2 diabetes and/or currently smoking patients. However, the above results confirmed the hypothesis that the prevalence of DEBs in schizophrenic patients is higher compared to the general population.

As the second part, no considerable difference was found in the EAT-26 scores based on gender. This finding is consistent with the results obtained by Kouidrat et al. [22], suggesting no significant gender difference in uncontrolled eating. However, a significantly higher risk of DEBs in the female gender was reported by Sallemi et al. [16] and Sentissi et al. [21]. Regardless of the results of Sallemi et al. [16] and Sentissi et al. [21], the present study together with wider literature proposed that DEBs were equally evident in men and women with schizophrenia, which is contrary to the general population that females are typically more affected [6]. Nevertheless, since EAT-26 is not specific to any FEDs (not even atypical or subthreshold ones), these findings should be used with caution.

As the third part, there were significant differences in the EAT-26 scores regarding the duration of psychosis and category of antipsychotic medications, which are consistent with the findings of Sallemi et al. [16], Sentissi et al. [21], Kouidrat et al. [6], Blouin et al. [18], and Fawzi et al. [34]. These results, however, are inconsistent with the results obtained by Kouidrat et al. [22] that showed no significant differences between TFEQ scores, category of antipsychotic medications, and duration of psychosis. These contradictory findings suggest that DEBs may represent a feature of schizophrenia regardless of medication use, such as antipsychotics [34]. Therefore, antipsychotic medication switching (as a strategy for reducing metabolic problems in schizophrenic patients) might not necessarily yield a better outcome [35]. In what follows, significant differences were observed in the EAT-26 scores regarding tobacco smoking and type 2 diabetes. These findings agree with those obtained by Essawy et al. [36] and García-Mayor et al. [26]. In this respect, tobacco smoking can increase the risk of internalizing, externalizing, and total behavioral problems (such as DEBs) by exacerbating interpersonal conflict and violence [25]. Further, in a similar direction, type 2 diabetes can raise the risk of developing DEBs due to dietary regimens, overweight, or obesity [26]. As an additional finding, the results of this study showed that psychosocial rehabilitation in schizophrenic patients was associated with a decrease in EAT-26 scores (discussed later).

As the fourth part, no significant difference was found in the EAT-26 scores concerning the phases of schizophrenia (active or remission phases). This finding was inconsistent with the results obtained by Khalil et al. [37], who demonstrated that DEBs decrease with psychotic episodes and recur when the psychotic episode remits. In this regard, DEBs may be a comorbid condition or part of the broad spectrum of psychotic disorders [8]. In other words, based on functional profiling of phenotypic manifestations, there may be a potential biologic overlap between subgroups of schizophrenia and DEBs. This caused DEBs to become phase-independent in some schizophrenic patients [8]. However, this hypothesis should be followed by a search for genetic correlates of variability.

As the fifth part, the results of the multiple linear regression analysis indicated that lack of psychosocial rehabilitation, use of atypical antipsychotics, an early stage of psychosis, high level of anxiety and depression, expression of more active psychotic symptoms, tobacco smoking, and type 2 diabetes were associated with increased development of DEBs among schizophrenic patients. These findings proposed that psychosocial rehabilitation accelerated the recovery from DEBs in schizophrenia. SAMHSA [38] defined recovery as “A process of change through which individuals improve their health and wellness, live a self-directed life, and strive to reach their full potential”. Accordingly, the following four domains support a recovering life: (1) health, i.e., overcoming and managing the disease while living in a physically and emotionally healthy manner; (2) home, i.e., a stable, constant, and safe place for living; (3) purpose, i.e., meaningful daily activities such as job, school, volunteerism, as well as creative endeavors, income, and resources, and (4) community, that involves communications and social networks providing support, friendship, love, and hope [38]. On this point, psychosocial interventions including Assertive Community Treatment (ACT), Supported Employment (SE), illness self-management training, family-based services, and Wellness Recovery Action Planning (WRAP) can mitigate stress and control DEBs in schizophrenic patients through rapid access to health services, medication management, job creation, as well as improvement in self-esteem, crisis intervention, emotional support, social interactions, and community reintegration. Furthermore, increasing the duration of psychosis through adaptation to a new reality of the illness can alleviate stress and, consequently, reduce overeating as an adaptive defense [39, 40]. However, it just might be a sign of the natural history of schizophrenia and FEDs—that FEDs usually happen earlier in life and schizophrenia is more prevalent later [41].

Concerning category of antipsychotic medications, the majority of the literature has suggested that uncontrolled eating behaviors are mostly influenced by treatment with atypical antipsychotics [18–21, 34]. This could partly explain the higher weight gain often reported in these patients in response to altered appetite sensations and increased susceptibility to hunger [18, 21]. On this detail, some mechanisms for gaining weight and increasing food intake related to antipsychotics are: (i) direct impacts on antipsychotics receptors; (ii) direct/indirect impacts on neuronal circuits (hypothalamus) that control satiety and food intake; (iii) disruption of the hypothalamic–pituitary–adrenal axis; (iv) direct influence on insulin secretion and sensitivity; (v) impacts on gastrointestinal hormones contained within food intake; (vi) diminished physical activity and reduced basal metabolism [6]. In fact, antipsychotic drugs are able to impact many neurotransmitter systems and take antagonistic actions on serotonin, dopamine, muscarinic, histamine, and adrenergic receptors [42]. All former neurotransmitters have had involvements (directly or indirectly) in the pathways related to regulating food intake [43], weight balance [44, 45], and metabolism [46, 47]. Blockades of serotonin (5HT2c), dopamine (D2 and D3), histamine (H1) [48], and muscarinic (M2 and M3) receptors have been found to raise appetite [49, 50]. Moreover, using endocrine/metabolic mechanisms, antipsychotics are able to directly trigger the hypothalamus–pituitary–adrenal axis activation [51], insulin secretion deficits [52], and gastrointestinal hormones changes [53]. Some other studies proposed a relationship between the antipsychotic drug-induced increased food intake and variations in melatonin, leptin, opioid, and endocannabinoid signaling [54].

As another finding, schizophrenic patients with higher total scores in PANSS were discovered with higher total scores in EAT-26. In other words, the presence of DEBs among schizophrenic patients was related to a more severe expression of active psychotic symptoms. In detail, this finding hypothesized that comorbidity of DEBs was associated with more severe schizophrenia psychopathology. Although there is very little evidence to support this finding, an explanatory model can be obtained from the dopamine hypothesis [55]. In fact, evidence implies that the dopaminergic system is a key mediator of appetitive conditioning [56].

Additionally, anxiety and depression were regarded as a risk factor for a variety of DEBs. On this matter, a recent hypothesis has suggested that DEBs may serve as a method whereby some subjects cope with anxiety and depression. In this regard, cognitive avoidance theory supports the role of anxiety and depression in the DEBs process by suggesting that patients with affective disorders engage in DEBs episodes to escape from this state [12]. The role of tobacco smoking and type 2 diabetes in the development of DEBs was previously discussed as well.

### Limitations of the study

This study has suffered some limitations. Firstly, the cross-sectional design avoided the precise understanding of relationships’ nature, especially causality. Next, self-reporting questionnaires cannot confirm a diagnosis, and it usually overestimates the prevalence of DEBs. It is possibly more prominent among schizophrenic patients, who may easily exaggerate or minimize the scorings. These limitations can be markedly resolved by designing longitudinal studies and interviewing individual participants. Thirdly, the results cannot be generalized to other regions since the sample size was limited to a single geographic region with unique individual, social, and cultural characteristics. Therefore, more extensive research needs to be carried out in other regions across the world. The fourth limitation is the absence of a standardized assessment concerning the study of DEBs among schizophrenic patients. EAT-26 has not been designed to make a diagnosis of any FEDs (not even atypical or subthreshold ones). Thus, it should not be used in place of any professional diagnosis as it has low positive predictive value due to denial and social desirability, as well as for the possible confounding role of comorbid factors [57]. So, it hampers an accurate evaluation of the frequency of DEBs.

## Conclusions

It is essential to identify DEBs among schizophrenic patients as an important cause of cardiometabolic disorders [6, 22]. Since DEBs occurrence is independent of different phases of schizophrenia, it becomes more critical to evaluate the risk of DEBs during the entire course of schizophrenia. Moreover, the use of psychosocial interventions, treatment of affective disorders (i.e., anxiety and depression), antipsychotic medication switching, treatment of tobacco smoking and type 2 diabetes may reduce the risk of DEBs among schizophrenic patients owing to the association between DEBs and psychosocial rehabilitation, category of antipsychotic medications, anxiety, depression, tobacco smoking, and type 2 diabetes. Additionally, the role of duration of psychosis in the development of DEBs indicated the need for a more precise examination of such behaviors at earlier stages of schizophrenia. Nevertheless, further studies (in particular longitudinal and empirical research) are required to prove the actual roles of the above factors in developing DEBs among schizophrenic patients.

## Data Availability

The datasets generated and analyzed during the current study are not publicly available because no consent was obtained from the participants in this regard. However, the data are available from the corresponding author on a reasonable request.

## Abbreviations

ACT: Assertive community treatment
ANOVA: Analysis of variance
BAI: Beck Anxiety Inventory
BDI-II: Beck Depression Inventory
DEBs: Disordered eating behaviors
DSM-5: Diagnostic and Statistical Manual of Mental Disorders, 5th Edition
EAT-26: The 26-item Eating Attitudes Test
EAT-40: The 40-item Eating Attitudes Test
FEDs: Feeding and eating disorders
GHQ-28: The 28-item General Health Questionnaire
PANSS: Positive and Negative Syndrome Scale
SCID-5-RV: Structured Clinical Interviews for DSM-5: Research Version
SE: Supported employment
TFEQ: Three-Factor Eating Questionnaire
WRAP: Wellness recovery action planning
